# The virgin lands campaign and the occurrence of foot-and-mouth disease and anthrax in the Republic of Kazakhstan (1955–1970)

**DOI:** 10.3389/fvets.2025.1549307

**Published:** 2025-06-02

**Authors:** Yersyn Y. Mukhanbetkaliyev, Ablaikhan S. Kadyrov, Maxat A. Berdikulov, Aizada A. Mukhanbetkaliyeva, Alimzhan S. Kadyrov, Assylbek A. Zhanabayev, Fedor I. Korennoy, Andres M. Perez, Sarsenbay K. Abdrakhmanov

**Affiliations:** ^1^Faculty of Veterinary and Livestock Technology, S. Seifullin Kazakh Agro Technical Research University, Astana, Kazakhstan; ^2^RSE National Veterinary Reference Center, Astana, Kazakhstan; ^3^Federal Centre for Animal Health (FGBI ARRIAH), Vladimir, Russia; ^4^Center for Animal Health and Food Safety, College of Veterinary Medicine, University of Minnesota, Saint Paul, MN, United States

**Keywords:** foot-and-mouth disease, anthrax, epidemiology, virgin lands campaign, Kazakhstan

## Abstract

The campaign for the development of virgin lands in Kazakhstan (1955–1970) was one of the most ambitious programs implemented by the Soviet government, which, arguably, resulted in both positive and negative consequences for the country. The campaign brought, at the same time, development, environmental degradation, and a dramatic cultural change to Kazakhstan. A barely explored aspect of the virgin lands campaign is related to its impact on the epidemiology of animal diseases. This paper describes, for the first time, the changes experienced by Kazakhstan during the implementation of the virgin lands campaign, offering a perspective on how those changes may have affected the occurrence of foot-and-mouth disease (FMD) and anthrax. Newly organized livestock premises and processing plants were created, which increased the concentration and intensification of animal production, in the absence of effective disease control plans. The initial increase in FMD prevalence may have been explained by the concentration of susceptible animals in the absence of appropriate control measures, followed by a decrease in disease incidence, probably explained by the enhancement of control measures associated with the formalization of livestock production, including improvements in vaccine quality. In contrast, soil degradation and the increase in the number of livestock, which resulted in a large number of animals buried in inappropriate conditions, may explain the sustained increase in the incidence of anthrax. The results presented here help to document the history of animal diseases in the country and ultimately contribute to the design of holistic strategies to support Kazakhstan’s development.

## 1 Introduction

The virgin lands campaign marked a significant turning point in the agricultural landscape of the Soviet Union. Launched in 1954 under the leadership of Soviet Premier Nikita Khrushchev, this ambitious program aimed to boost grain production and address food shortages by cultivating unproductive lands, predominantly in the vast steppes of Kazakhstan and parts of Siberia. The campaign was characterized by the mobilization of a massive workforce with the objective of converting the virgin soil into productive agricultural land (1, 2).

**Figure 1:**
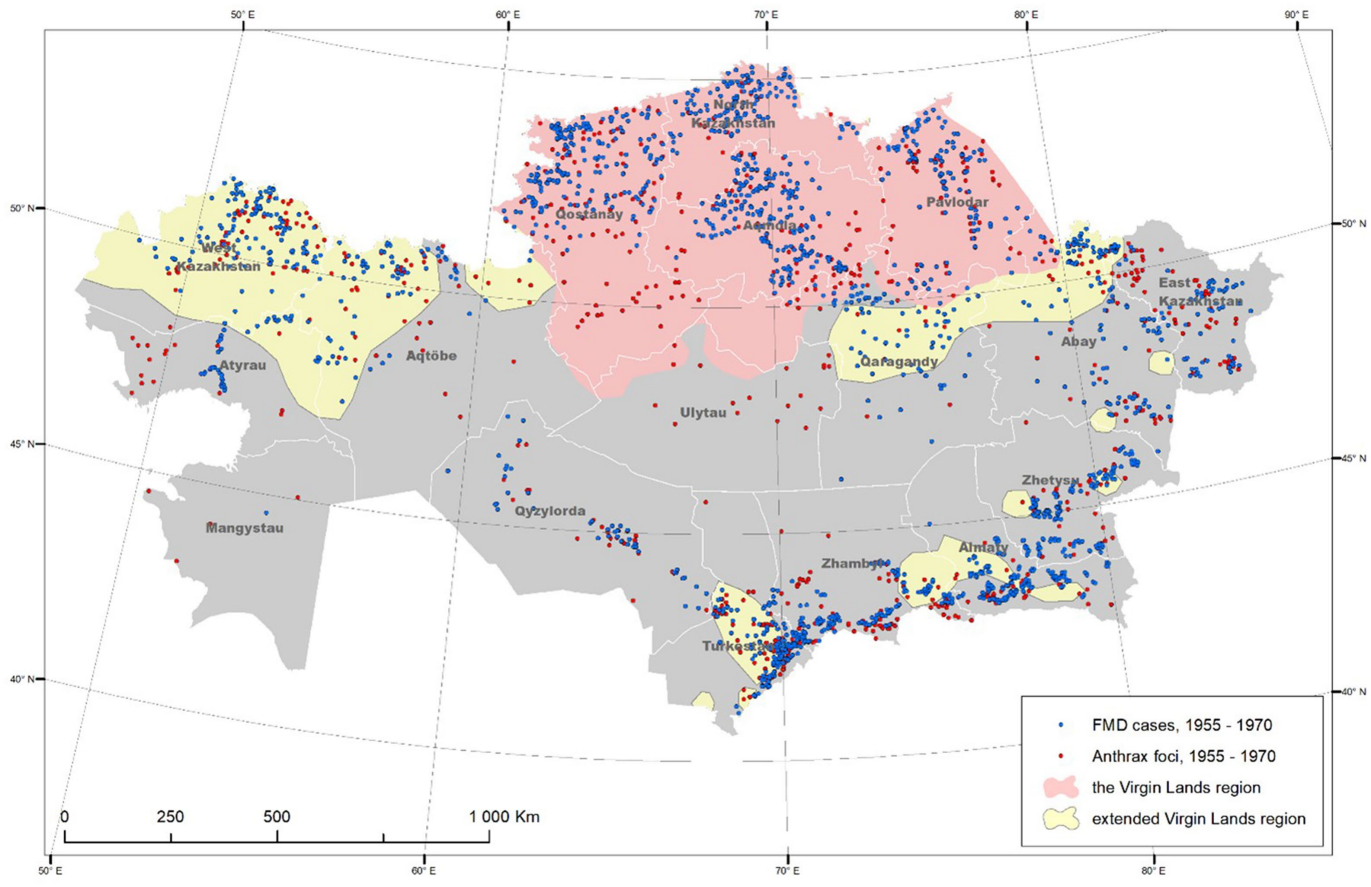
Geographical extension of the virgin lands campaign and distribution of foot-and-mouth disease and anthrax cases reported in the Republic of Kazakhstan during that period (1955–1970).

The motivation behind the virgin lands campaign was twofold, namely, to enhance national food security, and to assert Soviet dominance in agricultural production during the Cold War. By transforming the untouched steppe into cropland, the Soviet government sought not only to increase wheat yields but also to promote the ideological tenets of communism through the collectivization of agriculture (3, 4). For this purpose, approximately 25 million hectares of Kazakh steppe lands in Kokchetau (5 million hectares), Akmola (>4 million hectares), Kostanay (6 million hectares), Pavlodar (>2 million hectares), North Kazakhstan (~3 million hectares), and Turgay (>2 million hectares) were plowed up to turn them into fertile agricultural land (5) (Figure 1). This undertaking came with significant investments in infrastructure and technology, aiming to integrate Kazakhstan into the broader economic framework of the Soviet Union (6, 7).

Despite initial successes in terms of production output, the long-term impact of the virgin lands campaign on Kazakhstan’s agricultural sector was complex and often contradictory. Evaluation of the impact (positive or negative) was certainly influenced by political views (pro- or anti-communism) and such evaluation is out of the scope of this manuscript (1, 6, 8). It is generally accepted, however, that early reports heralded an increase in grain production, leading to improvements in food availability and development (1, 4, 8). However, these gains were often overshadowed by ecological, economic, and sociopolitical changes and challenges. The extensive land cultivation resulted in soil degradation, loss of biodiversity, and challenges related to water management and climate variability (3, 5, 9, 10).

One aspect relatively underexplored of this significant change is the impact of the virgin lands campaign on the occurrence of two diseases relevant to food animal production in Kazakhstan, namely, foot-and-mouth disease (FMD) and anthrax. This study, based on the analysis of archival records, seeks to provide insights into the long-lasting legacy of the virgin lands campaign on animal health and its relevance to current agricultural strategies in Kazakhstan. Understanding these dynamics is crucial not only for re-assessing the historical narrative of Soviet agricultural policies, but also for informing present-day approaches to sustainable land management and food security in the region.

## 2 Collectivization of food animal production in the Soviet Union

Collectivization of food animal production was implemented in the Soviet Union through the creation of two main types of farms, referred to as *kolkhozes* (or collective farms) and *sovkhozes* (or State farms), which operated under distinct organizational models, reflecting different approaches to organizing agriculture (11).

*Sovkhozes* were enterprises wholly owned and operated by the State and managed by appointed government agencies, which ensured centralized planning and control. State farm workers were government employees, which received a regular salary and with no sharing in production profits. State farms focused on the mass production of grain, meat, milk, and other agricultural products, which the state purchased at set prices. *Sovkhozes* were provided with machinery, fertilizers, and seeds from the state budget, which allowed them to operate at a higher level compared to small farms. In turn, they were expected to meet certain production volumes to supply the society (12, 13).

Conversely, *Kolkhozes* were associations of peasants who worked together on common land and shared the results of their labor. Managers were elected by the community, which implied a high degree of self-government, although they were still under government supervision. *Kolkhoz* members worked on their plots and in communal fields, and the results of their labor were distributed among the members of the collective farm depending on their contribution. *Kolkhozes* often grew a variety of crops and raised animals and, although production plans were still set up by the central government, their main objective was to ensure food security for their members and local markets (13, 14).

Although collectivization practices were launched in the late 1920s in the Soviet Union, policy was not intensively implemented in Central Asia until the virgin lands campaign incepted in 1955 (10, 15).

## 3 The virgin lands campaign

Before the virgin lands campaign, Kazakh steppes were a relatively untouched ecosystem. These vast areas were covered by natural vegetation such as feather grass and wormwood. Numerous species of wild animals and birds lived here, including saigas, bustards, marmots, and other species adapted to life in the open steppes. Soil erosion and depletion of water resources were minimal, because vegetation cover was sufficient (7, 11). Kazakhs were traditionally pastoral nomads that followed annual migration routes in search of pastures and lived in yurts. Sedentary food animal production at the beginning of the 20th century was dominated by traditional households and small farms focused on livestock raising and small-scale agriculture. Before 1954, the region contained ~1.1 million head of cattle, ~800 thousand sheep and goats, and ~80 thousand horses (5).

During the virgin lands campaign, 337 s*ovkhozes* were created in the Kazakh regions of Kokchetau (*n* ~ 90), Akmola (*n* ~ 80), Kostanay (*n* ~ 90), Pavlodar (*n* ~ 40), North Kazakhstan (*n* ~ 30), and Turgay (*n* ~ 10). Because of the incorporation of intensive management practices and financing, the number and density of farm animals increased substantially. By the beginning of the 1960s, Kazakhstani s*ovkhozes* engaged in virgin lands contained ~1.8 million head of cattle, ~1.5 million sheep and goats, and ~100 thousand horses. Perhaps more important than the increase in the number of food animals in the region was the changes in their density and housing practices (1, 8).

On the *sovkhozes*, farm animals were kept in conditions designed for mass production of by-products such as meat, milk, and wool. Conditions varied depending on the type of state farm and its specialization, but in general terms, production became much more intensive than in the past. New practices included stall housing (with animals kept indoors in winter), access to artificial pastures in summer, use of concentrated/balanced feed (including feed mills in certain farms), and veterinary care provided by the government. Genetically improved breeds were used and farm records (including production and health records, production plans, and record of management practices) were maintained (7, 13).

Intensification of food animal practices required of massive plowing of the steppes, which resulted in substantial environmental changes. On plowed lands deprived of natural vegetation, wind and water erosion increased (2, 3). The topsoil was destroyed, which led to a decrease in its fertility. As the soil degraded, crop yields decreased, which required increased costs for fertilizers and irrigation. The virgin lands campaign also led to a decrease in the populations of many species, as their natural habitat was destroyed (1, 9). For example, saigas, which migrated across the steppes, were forced out of these areas and their numbers significantly decreased. Some species of birds and small animals also disappeared. Intensive farming without proper irrigation and water management led to a decrease in groundwater levels. The aridity of the territories increased, especially in those areas where water was required for growing grain crops. After repeated use of land for grain crops without the introduction of crop rotation practices and measures to preserve soil fertility, productivity gradually decreased. In some regions, additional measures were required to restore the soil. Finally, mass plowing of the steppes led to a change in the landscape structure, which affected local climatic conditions such as wind speed and temperature (5, 10).

## 4 Impact on occurrence of foot-and-mouth disease (FMD) and anthrax

**Figure 2:**
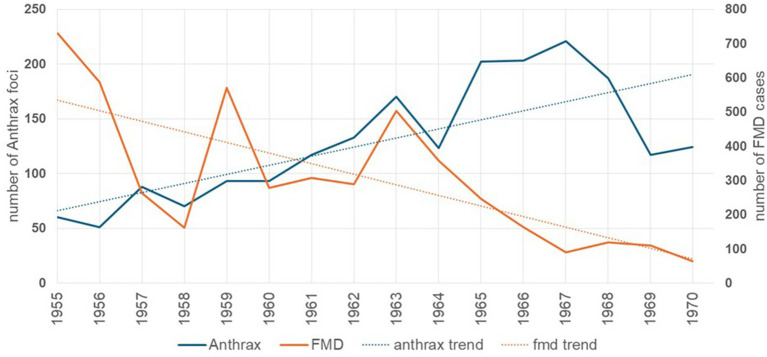
Annual number of cases (solid line) and linear trend (dashed line) of foot-and-mouth disease (orange lines) and anthrax (blue lines).

Between 1955 and 1991, FMD was reportable in the Soviet Union, and laboratory confirmation and serotype characterization were regularly conducted (16). Three main periods may be distinguished in the evolution of the disease in the country, of which the first period roughly matches the implementation of the virgin land campaign descried in this study (1955–1968). The period was characterized by an increase in the number of FMD-affected areas in the country. The virgin lands campaign was sustained by a massive migration (>2 million) of people from other regions of the Soviet Union, as well as a concomitant increase in the number of farm animals. The migration of the population was accompanied by migration of livestock, including virus carriers, which resulted in the increased probability of contact of susceptible animals with carriers of the virus. Also, an increase in the number of farm animals contributed to the expansion of pasture lands by covering the habitat of wild ruminants (potential carriers of the foot-and-mouth disease virus) (5). This contributed to direct and indirect contacts between domestic and wild ruminants, which consequently led to new outbreaks of infection. In addition, with the intensive development of the agro-industrial complex at that time, the transport infrastructure was actively developing. Therefore, the close economic and economic relations between farms provided by transport links also contributed to the manifestation of new outbreaks and the spread of infection to other territories. Another possible reason of the FMD incidence rise in this period was the epidemic caused by the introduction of the A22 virus from Iran. This new strain found a fertile landscape for spread through the newly created settlements, influenced in part by the high density and confinement of susceptible livestock, which favored disease spread (17). This 13-year period accounted for 80.5% of the outbreaks officially registered over 30 years in the country. Most (64%, *n* = 3,097) of the 4,835 FMD outbreaks reported in Kazakhstan occurred in areas of implementation of the virgin lands campaign (Figure 1). In the early 1960s, however, improvements in the development of FMD vaccines resulted in a sustained decline of disease incidence (18, 19). Overall, the initial increase in the number of outbreaks was followed by a significant (*p* < 0.01) linear decreasing trend in FMD incidence (Figure 2).

In turn, since 1955, a significant (*p* < 0.01) increase in the trend of annual incidence of anthrax was reported (Figure 2). In general, the period from 1954 to 1968 saw the highest rate of anthrax outbreaks registered in Kazakhstan. During this period, a total of 1,850 outbreaks of the disease were recorded, with an average of 123 outbreaks per year and a maximum peak of 221 outbreaks reported in 1967 (20, 21). One of the most likely reasons for this increase in the number of anthrax outbreaks was the extensive campaign for the development of virgin lands that began in 1954. Given the biology of the anthrax pathogen and its ability to persist for a long time in the soil at sites of unaccounted-for anthrax burials or animal deaths, especially in fertile soils rich in humus, large-scale arable work on virgin lands, large-scale construction, hydraulic engineering work undoubtedly influenced the dynamics of the epizootic process of the disease. The removal of the pathogen to the soil surface, contamination of environmental objects (soil, feed, water, machinery, etc.) by the pathogen, and wind dispersal of anthrax spores along with the soil over considerable distances undoubtedly increased the risk of infection of susceptible animals with anthrax spores. Along with the increase in population in virgin regions, new settlements (state farms and collective farms) were organized, where the number and density of susceptible animals increased accordingly. This situation led to an increase in the possibility of contact of susceptible animals with the source of infection, which consequently contributed to the emergence of new outbreaks of infection (22, 23). At the same time, although farm animals were vaccinated against anthrax since the early 1950s, vaccine coverage was insufficient. Reasons for the insufficient coverage of the vaccine included the intensive growth in the number of animals, insufficient provision of vaccines in the field, lack of personnel, and poor accounting and planning of veterinary measures (24). For those reasons, mass vaccination of all susceptible livestock against anthrax did not start until the 1960s. Consequently, most (52%, *n* = 1,061) of the 2,052 anthrax outbreaks reported during this period occurred in the areas of implementation of the virgin lands campaign.

## 5 Discussion

The virgin lands campaign was a major breakthrough in the history of the Soviet Union that changed the landscape of agricultural production in Kazakhstan. The intensification of food animal production resulted in an increase in the number and densities of livestock in vast areas of the country (1, 3). While increase of agricultural productivity is still nowadays an objective of many Central Asian countries, including Kazakhstan, the impact on animal diseases observed during the implementation of the virgin lands campaign demonstrate the need for considering ecological and environmental impacts when planning for such changes.

At the initial phase of the virgin land campaigns, FMD incidence was relatively high, whereas anthrax was relatively infrequent (Figure 1). Those figures may be explained by the increase in animal densities with no control on the status of animals at movement, which resulted in conditions that were favorable for the spread of airborne diseases, such as FMD (22, 25, 26). The gradual increase in intensification of the industry and increase of veterinary support, resulted in an enhancement of control and prevention practices, including the development of effective vaccines and improvement on biosecurity practices (27, 28). As a result of those improvements, FMD gradually decreased in the region. FMD control activities in Kazakhstan continued to be increasingly implemented in Kazakhstan and, 50 years after the end of the virgin lands campaign, the country is the only one in Central Asia that has reached FMD-free status (29, 30).

While much effort was put at the time on improving the intensification of livestock production, little attention was paid to the potential environmental impact of those changes. Animals were buried without sufficient planning, which resulted in the contamination of soil, favoring the survival and spread of diseases like anthrax (31–33).

In addition to the development of the virgin lands campaign, there were other factors impacting on the ecology and the environment during this period, which may have indirectly impact on the status of the diseases assessed here. In total, there were 8 large military test sites on the Kazakh territory, occupying more than 7 percent of its territory (34). The Semipalatinsk nuclear test site alone, located in the northeastern part of Kazakhstan, in a steppe and semi-desert zone, with a total area of 18,500 km2, caused an irreparable damage to environmental, public, and animal health. Over 40 years of nuclear testing (1949–1989), the Soviet military and scientists on the territory of the Semipalatinsk nuclear test site produced over 460 explosions of nuclear, thermonuclear and hydrogen weapons and thus, 2 million hectares of agricultural land were exposed to radioactive contamination (35, 36).

Also, the development of the mining and metallurgical industry in the Soviet period, together with the growth of economic indicators, led to environmental degradation and major environmental problems. Thus, a number of deposits of polymetals, rare earths and phosphorites in Kazakhstan contain uranium mineralization, which is extracted together with the main ore during mining. Some of the radioactive mineralization goes into dumps and tailings, some remains in the main products (especially in phosphorus fertilizers). In some coal deposits, the upper oxidized parts of coal seams are also accompanied by uranium mineralization. This coal is to be stored as radioactive waste. Today, as a result of the activities of enterprises of the mining and metallurgical complex, more than 20 billion tons of industrial waste have accumulated on the territory of Kazakhstan (36).

The development of agriculture is one of the important factors of economic growth in rural areas and has auxiliary and concomitant effects in the country. Kazakhstan has an important potential for the development of agriculture: the country ranks fifth in the world in terms of agricultural land area - almost 25 million hectares of arable land and more than 70 million hectares of pasture land (of which only 30% is currently used). The country has sufficient water resources, a relatively clean natural production base, opening up the opportunity for the production of high-quality products, proximity to large markets and significant growing investments in transport and trade corridors. The Government intends to use the significant untapped potential of agriculture (including animal husbandry) to create added value, develop exports, create jobs, and achieve inclusive and sustainable growth (37). The State actively supports this sector by providing concessional financing, subsidies and other measures to increase the competitiveness of agro-industrial products in domestic and foreign markets. The gross agricultural output of the Republic of Kazakhstan in the first half of 2024 reached 1.6 trillion tenge (approx. $3 billion). This growth is due to an increase in livestock production by 3.5% and in crop production by 3% (38).

At the same time, for the development of competitive, sustainable livestock export, the country needs important investments and reforms, both in the public and private sectors. A considerable growth of the number of publications devoted to the development of both large-scale and smallholder livestock breeding can be observed in the recent years (39–41).

Kazakhstan and other Central Asian countries is making very active efforts within the framework of the One Health concept aimed at improving the health of people, the environment, and animals. In 2022, Kazakhstan, together with the Kyrgyz Republic, Uzbekistan, Tajikistan and Turkmenistan, signed a joint protocol for the “Protection of food systems and prevention of pandemics in Central Asia,” giving an official start to the development of the One Health Action Framework in Central Asia. This program involves the development of investment principles aimed at achieving three priorities for Central Asian countries: preventing pandemics, strengthening the sustainability of food systems and agriculture, as well as developing regional trade and increasing competitiveness (42). Kazakhstan, like other Central Asian countries, faces significant regional challenges such as preventing and preparing for future pandemics, increasing the sustainability of food systems and preparing to take advantage of new opportunities, as well as addressing threats associated with the expansion of livestock production and the intensification of cross-border movement of animals and animal products. These common challenges are high on the agenda of Governments and require regional cooperation to develop prevention and control systems on a scale that cannot be implemented within a single country strategy. Toward that goal, it is essential to consider the lessons learned during the implementation of the virgin land campaign and implement an holistic approach that protects environmental, animal, and public health while promoting development.

## Data Availability

The datasets used in the study may be obtained from the corresponding author upon a reasonable request. Requests to access these datasets should be directed to Sarsenbay K. Abdrakhmanov, s_abdrakhmanov@mail.ru.
